# Simultaneous Inhibition of Rhamnolipid and Polyhydroxyalkanoic Acid Synthesis and Biofilm Formation in *Pseudomonas aeruginosa* by 2-Bromoalkanoic Acids: Effect of Inhibitor Alkyl-Chain-Length

**DOI:** 10.1371/journal.pone.0073986

**Published:** 2013-09-04

**Authors:** Merced Gutierrez, Mun Hwan Choi, Baoxia Tian, Ju Xu, Jong Kook Rho, Myeong Ok Kim, You-Hee Cho, Sung Chul Yoon

**Affiliations:** 1 Nano-Biomaterials Science Laboratory, Division of Applied Life Sciences, Graduate School, Gyeongsang National University, Jinju, Republic of Korea; 2 National Research Foundation Funded Pioneer Research Center for Alzheimer Disease Control, Gyeongsang National University, Jinju, Republic of Korea; 3 Neurobiology Laboratory, Division of Applied Life Sciences, Graduate School, Gyeongsang National University, Jinju, Republic of Korea; 4 Laboratory of Antiinfective Agents and Phage Therapy, College of Pharmacy, CHA University, Gyeonggi-do, Republic of Korea; The Scripps Research Institute and Sorrento Therapeutics, Inc., United States of America

## Abstract

*Pseudomonas aeruginosa,* an opportunistic human pathogen is known to synthesize rhamnolipid and polyhydroxyalkanoic acid (PHA) of which the acyl-group precursors (e.g., (R)-3-hydroxydecanoic acid) are provided through RhlA and PhaG enzyme, respectively, which have 57% gene sequence homology. The inhibitory effect of three 2-bromo-fatty acids of 2-bromohexanoic acid (2-BrHA), 2-bromooctanoic acid (2-BrOA) and 2-bromodecanoic acid (2-BrDA) was compared to get an insight into the biochemical nature of their probable dual inhibition against the two enzymes. The 2-bromo-compounds were found to inhibit rhamnolipid and PHA synthesis simultaneously in alkyl-chain-length dependent manner at several millimolar concentrations. The separate and dual inhibition of the RhlA and PhaG pathway by the 2-bromo-compounds in the wild-type cells was verified by investigating their inhibitory effects on the rhamnolipid and PHA synthesis in *P. aeruginosa* Δ*phaG* and Δ*rhlA* mutants. Unexpectedly, the order of inhibition strength was found 2-BrHA (≥90% at 2 mM) > 2-BrOA > 2-BrDA, equally for all of the rhamnolipids and PHA synthesis, swarming motility and biofilm formation. We suggest that the novel strongest inhibitor 2-BrHA could be potentially exploited to control the rhamnolipid-associated group behaviors of this pathogen as well as for its utilization as a lead compound in screening for antimicrobial agents based on new antimicrobial targets.

## Introduction

*Pseudomonas aeruginosa* is a typical opportunistic human pathogen which colonizes the lungs of cystic fibrosis patients and causes serious infections in immuno-compromised hosts [1]. It can simultaneously produce two biotechnologically important compounds, namely polyhydroxyalkanoic acids (PHAs) and rhamnolipids [2]. PHAs, which are promising materials for biodegradable plastics, have been studied extensively as replacements for conventional petrochemical-based plastics [3]. The Rhamnolipids, which represent one of the most important classes of microbial surfactants, are of increasing industrial interest because of their broad range of potential applications including use as surface coatings and also additives for environmental remediation [4], [5]. They serve as extracellular virulence factors that play multiple roles [4]–[6]. For example, they enhance uptake of hydrophobic substrates in an energy-dependent manner [7], display antibiotic activities, and contribute to pathogenesis [8]–[10]. Along with its precursor, β-hydroxyalkanoyl-β-hydroxyalkanoic acid (HAA) in which β-hydroxydecanoic acid (C_10_) is the major component, rhamnolipids have been demonstrated to play a central role in swarming motility [11]–[14].They are also implicated in various steps of biofilm development [15]–[19].

**Figure 1:**
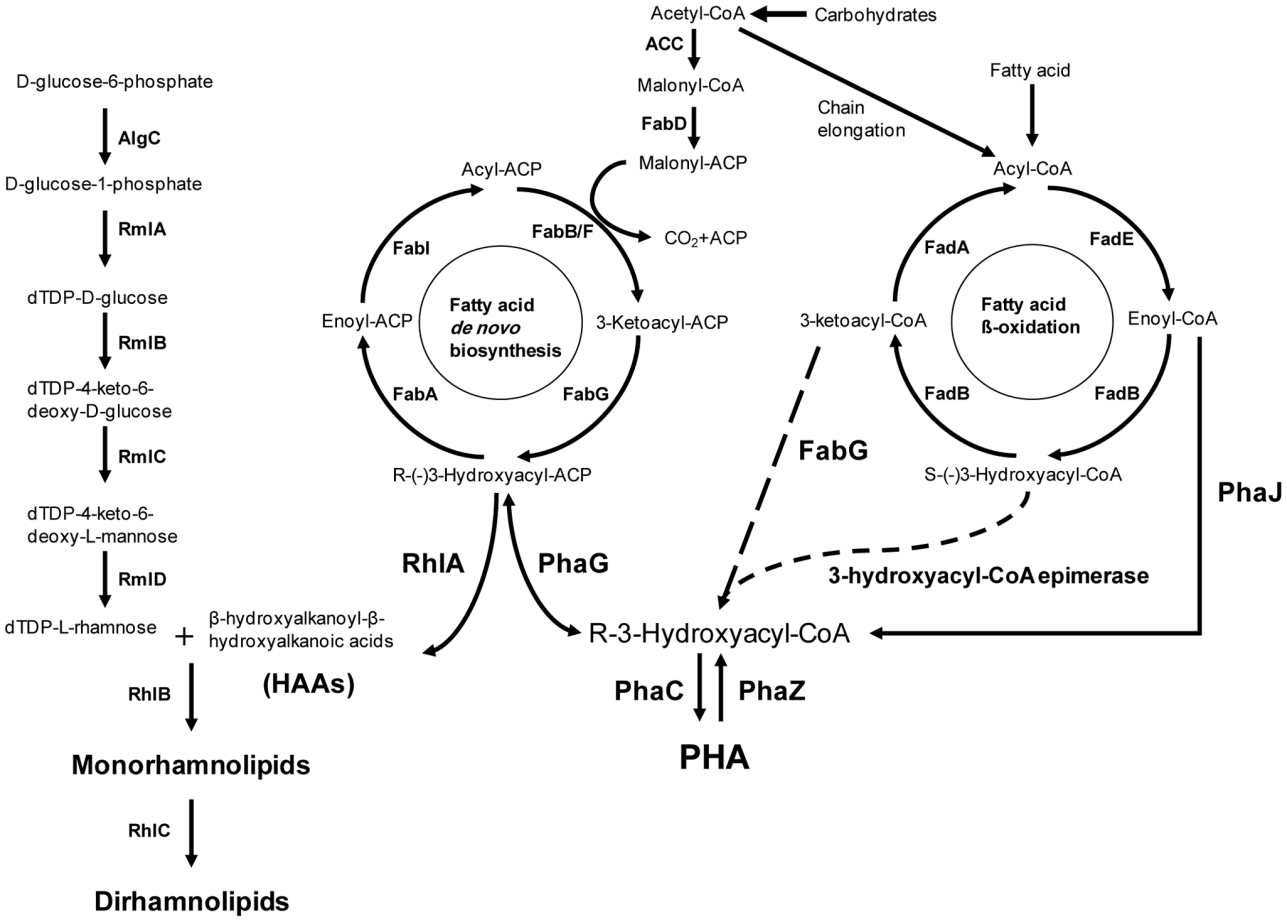
Parallel linked RhlA and PhaG metabolic pathways *via* fatty acid *de novo* biosynthesis leading to the synthesis of rhamnolipids and PHA. RhlA and PhaG (their amino acid sequences have 41% identity) are considered to be the targets of 2-bromo-inbibitors.

Two types of rhamnolipids are known: the monorhamnolipids (Rha-C_10_-C_10_), which contain one unit of rhamnose linked to HAA, and the dirhamnolipids (Rha-Rha-C_10_-C_10_), which contain two units of rhamnose (Figure 1) [9]. When *P. aeruginosa* is grown on glycerol and saccharides, (R)-β-hydroxyalkanoyl-acyl carrier protein ((R)-β-hydroxyalkanoyl-ACP) is utilized by RhlA (HAA synthase) to produce HAAs from two molecules of (R)-β-hydroxyalkanoyl-ACP [5], [12], [20]. In medium-chain-length (MCL, 6–14 carbon atoms)-polyhydroxyalkanoic acid (PHA) producing bacteria, such as *Pseudomonas* spp. belonging to rRNA group I, MCL-type (R)-β-hydroxyalkanoyl monomers are derived as the form of (R)-β-hydroxyalkanoyl-coenzyme A (CoA) which is the substrate of MCL-PHA synthase. The coenzyme A monomer is derived from ACP intermediates of the fatty acid *de novo* synthesis pathway via the enzyme (R)-β-hydroxyalkanoyl-ACP:CoA transacylase (PhaG) [21]. Thus, PhaG and RhlA may compete for (R)-β-hydroxyalkanoyl-ACP, especially (R)-β-hydroxydecanoyl-ACP which is the major acyl component of rhamnolipid [20]. However, it has been suggested that RhlA can produce CoA-linked fatty acid dimers using ACP-linked fatty acids [22], [23] and could also contribute to PHA synthesis by the RhlA activity which is analogous to that of PhaG. This suggestion is based on the fact that PHA synthesis in *P. aeruginosa phaG* mutants is not completely abrogated and *phaG* mutants of other *Pseudomonas* spp. completely lack PHA production when grown with a sugar as the carbon source. The gene *rhlB*, which encodes a rhamnosyltransferase, is known to be responsible for the synthesis of rhamnolipids by transferring a rhamnosyl group to HAA [5]. The gene *rhlC* encodes the rhamnosyltransferase II responsible for the addition of the second rhamnosyl group to the monorhamnolipid [5]. The close metabolic relationship between PHA and rhamnolipid synthesis was experimentally confirmed on the basis of comparative ^13^C NMR analysis of them in wild-type and mutants [24]. Higher PHA accumulation was found in the rhamnolipid-negative mutants than in the wild-type strains, suggesting that 3-hydroxy fatty acid precursors become more available for PHA synthesis when rhamnolipid synthesis is lacking. However, compared to the wild-type strains, rhamnolipid production was not enhanced in the four *pha* mutants of *P. aeruginosa* PA14 and PAO1 which indicates that rhamnolipid production in *P. aeruginosa* could be tightly regulated. This may be ascribable to transcriptional level regulation by a quorum-sensing (QS) response, since *P. aeruginosa* possesses two interrelated QS systems, (*las* and *rhl*) [25] that regulate rhamnolipid expression governed by three QS molecules: *Pseudomonas* autoinducer 1(PAI-1)[N-(3-oxododecanoyl) homoserine lactone also known as 3-oxo-C_12_-HSL] [26], *Pseudomonas* autoinducer 2 (PAI-2) [N-butyryl homoserine lactone known also as C_4_-HSL][27], and *Pseudomonas* Quinolone Signal (PQS) [2-heptyl-3-hydroxy-4-quinolone] [28].

PHA synthesis inhibitors have been used to find the metabolic pathway from which precursors for PHA synthesis are supplied, as well as to channel intermediates of a pathway specific to PHA synthesis [29]–[33]. In previous inhibitor screening studies, we reported that 2-bromooctanoic acid (2-BrOA) inhibits MCL-PHA synthesis by *Pseudomonas fluorescens* BM07 from fructose, without any influence on cell growth [32], [33]. It was suggested that 2-BrOA might specifically inhibit the enzyme PhaG. The genome sequence of *P. aeruginosa* PA14 showed that RhlA and PhaG had about 57% sequence homology. The probable substrate competition between RhlA and PhaG for (R)-β-hydroxyalkanoyl-ACP and their high sequence homology may suggest that RhlA-mediated rhamnolipid synthesis could also be inhibited by 2-BrOA. Here, we investigated the inhibitory effect of two more 2-bromoalkanoic acids, 2-bromohexanoic acid (2-BrHA) and 2-bromodecanoic acid (2-BrDA) as well as 2-BrOA in *P. aeruginosa* strains PA14 and PAO1 on the synthesis of rhamnolipids and PHA to investigate the nature of the dual inhibition by 2-bromo-substituted compounds as a function of their alkyl-chain-length. In addition, their relevant effects on swarming motility and biofilm formation were also investigated.

## Materials and Methods

### Bacterial strains

*P. aeruginosa* PA14 wild-type and PA14-Δ*phaG* mutant were obtained from PA14 NR set [24]. *P. aeruginosa* PAO1 wild-type and PAO1-Δ *rhlA* mutant were purchased from the University of Washington Transposon Mutant Collection [24]. Nutrient-rich (NR) medium was used in seeding, maintenance, and storage of the bacterial strains and contained 1% yeast extract, 1.5% nutrient broth, and 0.2% ammonium sulfate. The modified M1 mineral salts medium of the same composition as that reported earlier [24] was used as the medium for PHA and rhamnolipid synthesis. Antibiotics were added to growth media in the following concentrations: tetracycline, 60 µg/ml; gentamicin, 30 µg/ml.

2-BrHA (98% purity, GR grade) and 2-BrOA (97% purity) were purchased from Tokyo Chemical Industry Co., LTD and 2-BrDA (96% purity) from Fluka Analytical.

### Cell growth monitoring

The culture (5 ml) grown in NR medium at 30°C at 180 rpm for 12 h was transferred to 500 ml of M1 medium containing 70 mM fructose and 1.0 g of ammonium sulfate/liter in a 2-L flask and aerobically cultivated until a steady-state growth was reached. The cell growth was determined by measuring dry cell weight (DCW). The cells were isolated by centrifugation (10,000×g, 10 min) of the culture, washed with methanol, and dried under vacuum at room temperature for 24 h. The concentration of remaining fructose and ammonium ion in the media was measured by using the 3,5-dinitrosalicylic acid (DNS) method and Nessler’s reagent method respectively [33].

### Rhamnolipid assay

Rhamnolipid agar plates were prepared by modifying a previously described protocol [13], [34]. The medium composition was based on M9 salts supplemented with 0.2% fructose, 2 mM MgSO_4_, 0.0005% methylene blue, 0.02% cetyltrimethylammonium bromide, and 2 mM 2-bromo-compound (if required) and solidified with 1.8% agar. Glutamate, used as the sole nitrogen source was added to a final concentration of 0.05%. After solidification, plates were inoculated by sterile toothpick with individual colonies from a fresh Luria-Bertani (LB) agar plate. Plates were incubated at 30°C for 24 h and then kept for at least 48 h at room temperature until a blue halo appeared around the colonies, indicating the production of rhamnolipid [34]–[37]. The orcinol assay [38] was used to directly assess the amount of rhamnolipids secreted into medium during cultivation on the modified M1 mineral-salts medium. Three-hundred microliter of the culture supernatant was extracted twice with 600 µl of diethyl ether. The ether fractions were pooled and evaporated to dryness, and 100 µl H_2_O was added. To 100 µl of each sample, 100 µl of an aqueous 1.71% orcinol solution and 800 µl 60% (v/v) H_2_SO_4_ were added; after heating for 30 min at 80°C, the samples were cooled for 15 min at room temperature and the absorbance was measured at 421 nm. The concentrations of rhamnolipids were indicated by comparing the data with those obtained with rhamnose standards between 0 and 50 mg/ml. The acyl-group composition of rhamnolipid was determined by gas chromatographic (GC) analysis. The supernatant of each culture was recovered after harvesting the cells, which were then freeze-dried and extracted with chloroform. The filtered chloroform extract was concentrated using a rotary evaporator (Eyela N-1000, Japan). An aliquot of the chloroform extract was subjected to a methanolysis reaction followed by GC analysis as described below.

### Quantitative assay of PHA in cells

For the analysis of PHA in cells, 20 mg of dried cells were reacted with a mixture containing 1 ml of chloroform, 0.85 ml of methanol and 0.15 ml of concentrated sulfuric acid. The reaction mixture in a closed screw capped tube was incubated at 100°C for 3 h and the organic layer containing the reaction products was separated, dried over MgSO_4_ and analyzed by gas chromatography. Each peak was standardized against standard 3-hydroxy-methylesters which were obtained by methanolysis of purified PHA with known compositions, determined by quantitative NMR analysis [33]. Gas chromatograms were obtained on a Hewlett Packard 5890A gas chromatograph equipped with a HP-5 capillary column (5%diphenyl-95% dimethyl-polysiloxane, 30 m ×0.535 mm i.d., 2.65 µm film thickness, J&W Scientific, Agilent Technologies, Palo Alto, CA, USA) and a flame ionization detector. The column was heated at 10°C/min from 80 to 250°C [29].

### Swarming motility assay

Swarm agar was based on modified M9 salts medium [13] supplemented with 1 mM MgSO_4_, 0.2% glucose, 0.05% glutamate as the sole nitrogen source, and 2 mM 2-bromo-compound (if required) and solidified with 0.6% agar. Cells were inoculated with 2 µl of an overnight LB culture into the middle of the swarm agar plates. Swarm agar plates were incubated for 16 h at 37°C and then incubated for an additional 48 h at 30°C.

### Biofilm formation assay

Biofilm formation was determined using a protocol modified by O’Toole and Kolter [39]. Cells were grown in 4 ml of M1 medium with 70 mM fructose in the presence or absence of 5 mM 2-bromo-compound at 30°C in glass tubes without agitation for 24 and 48 h. Static biofilm formation was measured by visual inspection of the air-liquid interface of the cultures. Coverage of the air-liquid interface of the culture by a layer of cells and matrix material was considered a biofilm. The tubes were washed with distilled water and stained with 0.1% crystal violet solution for 20 min. After addition of 4.5 ml of 95% ethanol to each tube, the adsorbed dye was quantified from the OD readings at 600 nm.

### Quantitative real-time PCR analysis of *phaG* and *rhlA* gene expression

RNA isolation was performed by using the RNeasy mini kit according to the manufacturer’s protocol. One-step RT-PCR was applied, using the oligonucleotide primer pairs for the *phaG* and *rhlA* gene and the Rotor-Gene SYBR Green RT-PCR kit. rRNA (16S) was used as an internal standard to estimate the relative expression level of the target gene. Cells were grown in M1 medium with 70 mM fructose in the presence or absence of 2 or 5 mM 2-bromo-compound at 30°C for 72 h.

### Assay of 2-bromoalkanoic acids remaining in the media

The 2-bromo-compounds remaining in the culture supernatant and cell pellets were extracted with chloroform and the chloroform extract was reacted in a sulfuric acid/methanol mixture. The methyl ester in the organic layer was analyzed using a Hewlett Packard 5890A gas chromatograph equipped with a HP-1capillary column (poly(dimethylsiloxane), 30 m×0.535 mm i.d., 2.65 µm film thickness, J&W Scientific, Agilent Technologies, Palo Alto, CA, USA) and a flame ionization detector. Typical GC run conditions were as follows: initial temp, 80°C, 2 min; heating rate, 10°C/min; final temp, 250°C, 1.75 min; carrier gas (He) constant flow rate, 3.1 ml/min; injector temp, 230°C; detector temp, 280°C.

## Results and Discussion

### Dual inhibitory effect of 2-bromoalkanoic acids on rhamnolipid and PHA production

**Figure 2:**
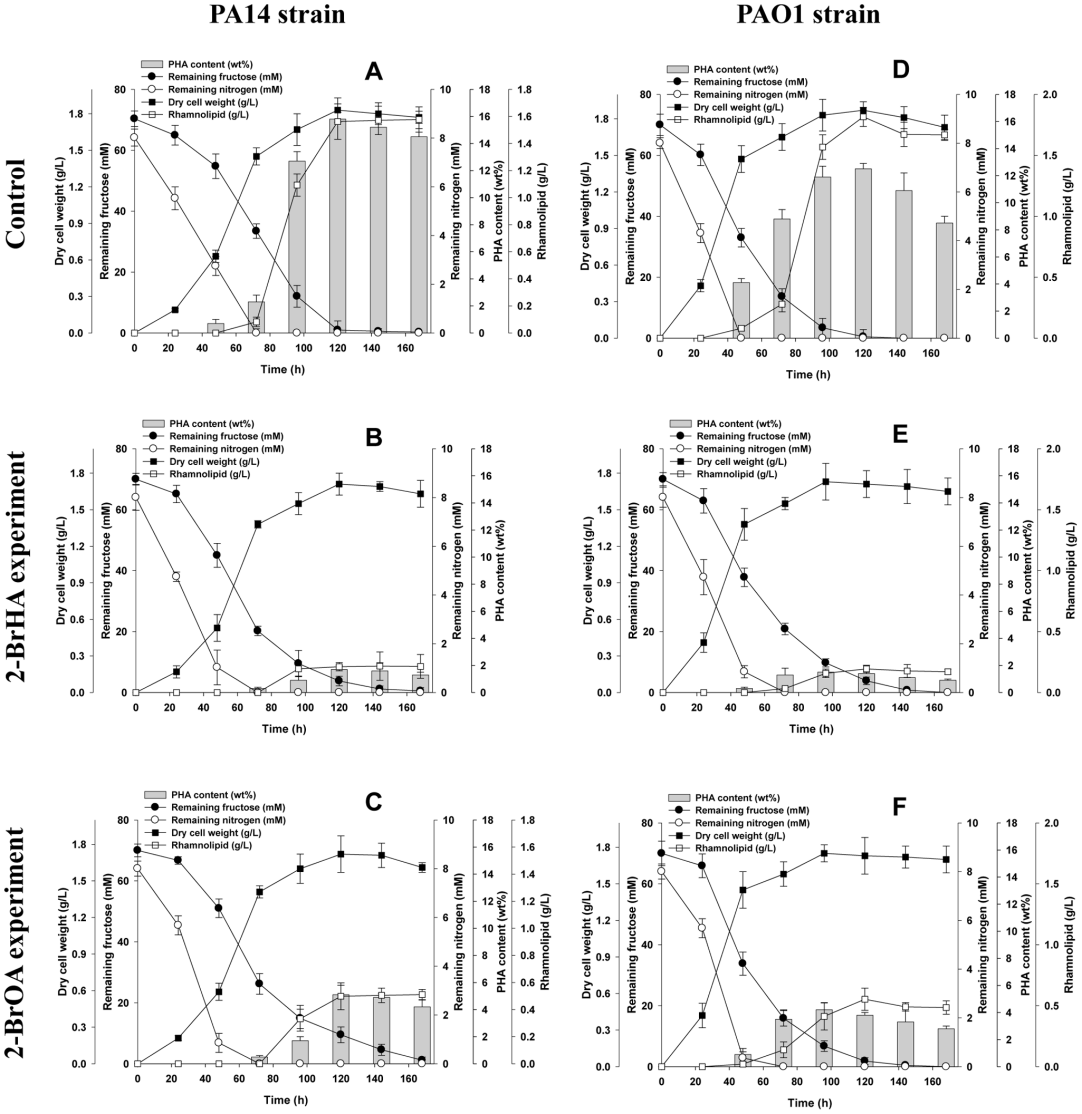
Effect of alkyl chain length of the 2-bromo- inhibitors on the time course profiles. (A), (B) and (C) represent the *P. aeruginosa* PA14 strain, and (D), (E) and (F) represent the *P. aeruginosa* PAO1 strain, both of which were grown aerobically in 70 mM fructose M1 medium without (A and D) or with (B and E) 2 mM 2-BrHA or (C and F) 2 mM 2-BrOA.

*P. aeruginosa* strains, PA14 and PAO1 were cultured in M1 medium containing 70 mM fructose. Cell growth, PHA and rhamnolipid production occurred in a growth-associated fashion. Active rhamnolipid secretion occurred during the active PHA accumulation phase when the nitrogen was almost depleted (Figure 2A for the PA14 strain and Figure 2D for the PAO1 strain). Maximal PHA accumulation was observed in the late stationary phase when the carbon source was exhausted. After exhaustion of the carbon source, PHA content decreased while rhamnolipid synthesis continued.

**Figure 3:**
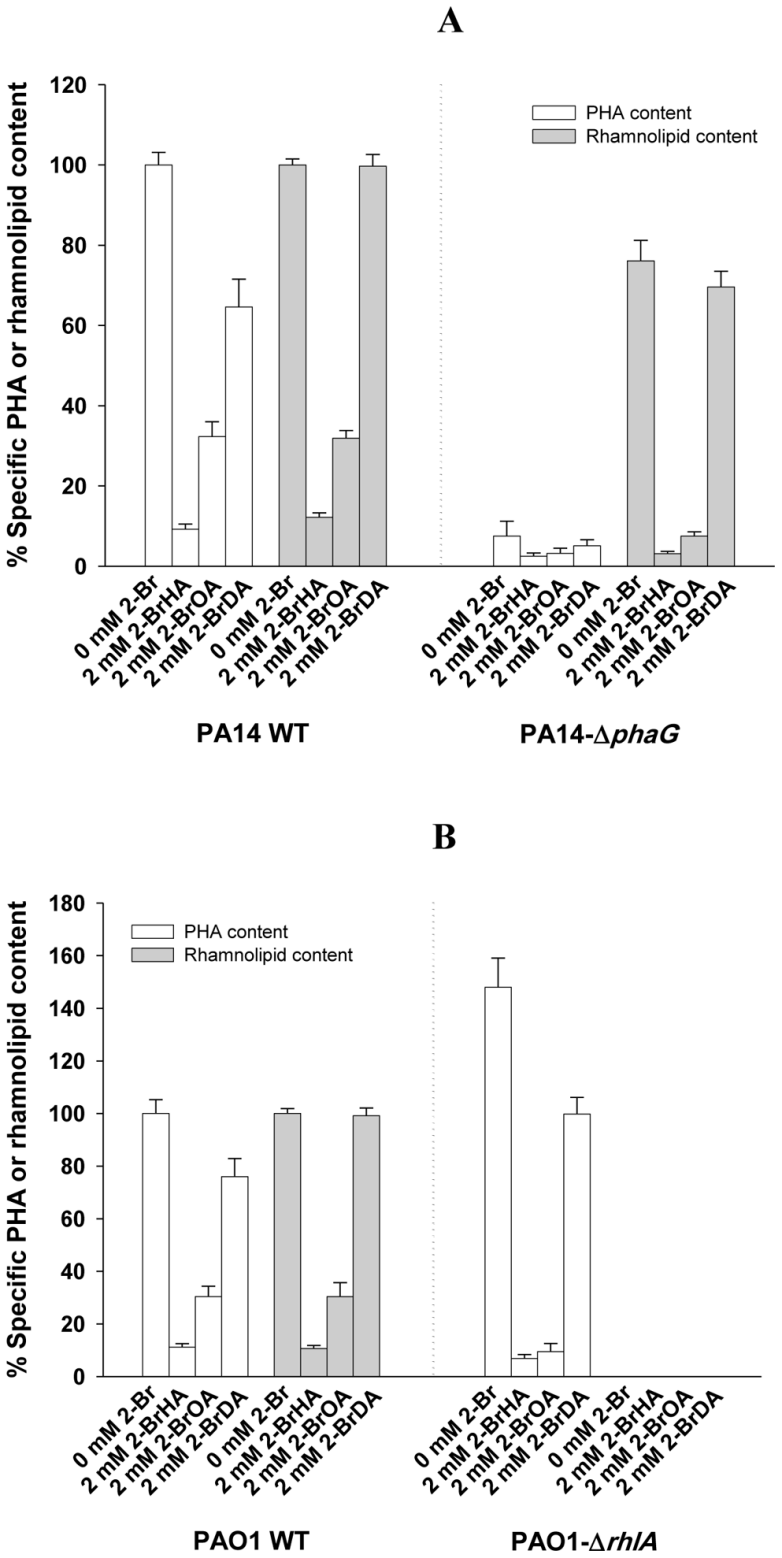
Separate inhibition of the PhaG and RhlA pathways by the 2-bromo- inhibitors in their respective mutant strains. Effect of the 2-bromo- inhibitors on PHA and rhamnolipid synthesis in *P. aeruginosa* PA14 and PA14-Δ*phaG* (A) and *P. aeruginosa* PAO1 and PAO1-Δ*rhlA* (B). The cells were grown in 70 mM fructose M1 medium without the 2-bromo-compounds or with 2 mM 2-BrHA, 2 mM 2-BrOA or 2 mM 2-BrDA.

The effect of the three 2-bromo-compounds (2-BrHA, 2-BrOA and 2-BrDA) on the PHA and rhamnolipid synthesis in *P. aeruginosa* strains PA14 and PAO1 was investigated (Table 1). The growth curve for *P. aeruginosa* cells grown on 70 mM fructose medium with 2 mM 2-BrHA is shown in Figure 2B and E. Generally, addition of 2 mM 2-bromo- compounds did not affect the growth of the two strains. However the addition of the inhibitors suppressed the maximum production of PHA and rhamnolipid in the PA14 strain from 16 wt% (at 120 h of cultivation) and 1.56 g/l (at 168 h of cultivation) to 1.7 wt% and 0.19 g/l for 2-BrHA, 5.1 wt% and 0.50 g/l for 2-BrOA and 10.2 wt% and 1.56 g/l for 2-BrDA, respectively (Table 1, Figure 2B and C). Similar decreases in production depending on the type of inhibitor were observed for *P. aeruginosa* PAO1 grown on 70 mM fructose medium with 2 mM 2-bromo-compounds: from 13 wt% (at 120 h of cultivation) and 1.82 g/l (at 168 h of cultivation) to 1.4 wt% and 0.19 g/l for 2-BrHA, 3.8 wt% and 0.55 g/l for 2-BrOA and 9.5 wt% and 1.80 g/l for 2-BrDA, respectively (Table 1, Figure 2E and F). The calculated % inhibition of PHA and rhamnolipid synthesis for the PA14 strain showed that the shortest alkyl-chain compound, 2-BrHA is the strongest inhibitor: 89.3%, 2-BrHA; 67.8%, 2-BrOA, and 35.5%, 2-BrDA for PHA synthesis; 87.8%, 2-BrHA; 68.2%, 2-BrOA, and 0.3%, 2-BrDA for rhamnolipid synthesis (Figure 3A). A similar type of inhibition was observed for the PAO1 strain (Figure 3B). Thus, for both strains, 2-BrHA and 2-BrOA exhibited similar levels of suppression of the production of PHA and rhamnolipids, respectively, whereas suppression by 2-BrDA elicited a different response: PHA production was inhibited by 25∼35%, but rhamnolipid synthesis was not affected by 2-BrDA (Figure 3A and B). 2-BrHA and 2-BrOA caused rather significant changes in the PHA monomer composition with 3-hydroxydecanoate as the predominant monomer. This was observed only for the PA14 strain but not for the PAO1 strain (68 mol% for the PA14 control and 52 mol% for 2-BrHA and 2-BrOA treated PA14) at maximum PHA accumulation. However, 2-BrDA treatment caused little change in PHA composition. The acyl group composition of the rhamnolipid was relatively constant and independent of cultivation time and 2-bromo- compound addition (Table 1). These results indicate that 2-BrHA and 2-BrOA inhibit both PHA and rhamnolipid synthesis in *P. aeruginosa* PA14 and PAO1 grown with fructose, with 2-BrHA having a more pronounced effect than 2-BrOA.

**Table 1:** Effect of 2-bromohexanoic acid (2-BrHA), 2-bromooctanoic acid (2-BrOA), and 2-bromodecanoic acid (2-BrDA) on PHA and rhamnolipid synthesis in *P. aeruginosa* PA14 and PAO1 grown with 70 mM fructose under one-step cultivation conditions at 30°C.

Strain	Co-added compounds (mM)	Culture time (h)	Dry cell weight (g/L)	PHA content (wt%)	PHA monomer-unit composition (mol%)^a^			Rhamnolipid content (g/L)	Rhamnolipid acyl-group composition (mol%)^a^			
					3HC^b^	3HO	3HD		3HO^b^	3HD	3HDDe	3HDD
**PA14**	No inhibitor	72	1.45±0.07	2.3±0.5	4.0±0.3	26.1±0.5	69.9±0.8	0.082±0.035	10.0±0.9	87.5±0.7	1.1±0.1	1.4±0.1
		96	1.67±0.13	12.7±0.7	4.5±0.5	27.2±0.9	68.3±0.4	1.093±0.082	10.2±0.6	87.3±0.4	1.0±0.1	1.5±0.1
		120	1.83±0.10	15.8±0.5	4.5±0.2	27.3±0.5	68.2±0.7	1.562±0.131	10.5±0.1	86.4±0.3	1.4±0.1	1.7±0.1
		144	1.80±0.09	15.2±0.4	4.3±0.3	27.6±0.7	68.1±0.4	1.571±0.105	10.4±0.5	86.7±0.2	1.3±0.1	1.6±0.2
	2 mM 2-BrHA	72	1.38±0.03	0.3±0.1	5.0±0.3	41.7±0.5	53.3±0.2	-c	-c	-c	-c	-c
		96	1.55±0.09	0.9±0.3	5.0±0.5	42.9±0.6	52.1±0.1	0.175±0.053	8.1±0.4	89.4±0.6	1.1±0.1	1.4±0.1
		120	1.71±0.09	1.7±0.3	5.2±0.5	43.3±0.2	51.5±0.3	0.191±0.031	8.5±0.3	88.4±0.1	1.4±0.1	1.7±0.1
		144	1.69±0.04	1.6±0.6	5.2±0.2	43.7±0.7	51.1±0.5	0.195±0.105	8.4±0.6	88.5±0.3	1.4±0.1	1.7±0.2
	2 mM 2-BrOA	72	1.41±0.05	0.5±0.1	5.0±0.3	40.5±0.2	54.5±0.5	-c	-c	-c	-c	-c
		96	1.60±0.12	1.7±0.3	5.1±0.5	42.1±0.6	52.8±0.1	0.332±0.071	8.2±0.4	89.2±0.2	1.1±0.1	1.5±0.1
		120	1.72±0.15	5.1±0.7	4.9±0.1	42.9±0.6	52.2±0.7	0.498±0.099	8.5±0.2	88.4±0.5	1.4±0.1	1.7±0.2
		144	1.71±0.10	4.9±0.3	4.8±0.3	43.4±0.3	51.8±0.6	0.505±0.053	8.5±0.5	88.6±0.2	1.3±0.1	1.6±0.2
	2 mM 2-BrDA	72	1.42±0.10	1.5±0.3	4.0±0.3	24.1±0.5	71.9±0.8	0.077±0.025	9.5±0.5	88.0±0.7	1.1±0.1	1.4±0.1
		96	1.63±0.11	8.1±0.7	4.1±0.5	26.6±0.9	69.3±0.4	0.991±0.055	9.7±0.6	87.6±0.4	1.2±0.1	1.5±0.1
		120	1.80±0.06	10.2±0.5	4.3±0.2	27.0±0.5	68.7±0.7	1.558±0.130	10.0±0.1	86.9±0.4	1.4±0.1	1.7±0.2
		144	1.77±0.13	10.6±0.7	4.3±0.3	27.3±0.7	68.4±0.4	1.563±0.123	10.0±0.3	86.9±0.6	1.4±0.1	1.7±0.2
**PAO1**	No inhibitor	72	1.65±0.11	8.8±0.7	5.2±0.3	34.8±0.7	60.0±0.4	0.279±0.063	9.6±0.2	87.6±0.5	1.3±0.1	1.5±0.2
		96	1.83±0.13	11.9±0.8	5.9±0.2	36.1±0.7	58.0±0.4	1.568±0.101	9.8±0.7	87.2±0.5	1.4±0.1	1.6±0.1
		120	1.87±0.07	12.5±0.4	6.3±0.2	36.6±0.5	57.1±0.3	1.816±0.079	9.7±0.3	87.2±0.6	1.4±0.1	1.7±0.2
		144	1.81±0.09	10.9±1.3	6.1±0.5	36.6±03	57.3±0.2	1.672±0.099	9.9±0.5	87.0±0.3	1.4±0.1	1.7±0.1
	2 mM 2-BrHA	72	1.55±0.05	1.3±0.5	5.0±0.1	33.5±0.3	61.5±0.4	0.033±0.012	9.3±0.2	87.9±0.5	1.3±0.1	1.5±0.2
		96	1.73±0.15	1.5±0.1	5.5±0.3	33.7±0.7	60.8±0.4	0.158±0.035	8.8±0.7	88.2±0.5	1.4±0.1	1.6±0.1
		120	1.71±0.11	1.4±0.3	6.1±0.2	35.1±0.5	58.8±0.3	0.194±0.029	8.9±0.1	88.0±0.4	1.4±0.1	1.7±0.2
		144	1.69±0.14	1.1±0.5	6.1±0.5	35.3±0.4	58.6±0.1	0.177±0.053	8.9±0.3	88.0±0.6	1.4±0.1	1.7±0.2
	2 mM 2-BrOA	72	1.58±0.10	3.5±0.7	5.6±0.5	33.5±0.2	60.9±0.3	0.138±0.065	8.7±0.2	88.5±0.5	1.3±0.1	1.5±0.2
		96	1.75±0.07	4.2±0.5	5.9±0.3	34.3±0.7	59.8±0.4	0.413±0.112	8.6±0.6	88.4±0.4	1.4±0.1	1.6±0.1
		120	1.73±0.15	3.8±0.2	6.1±0.3	34.8±0.6	59.1±0.3	0.553±0.091	8.5±0.3	88.5±0.6	1.4±0.1	1.6±0.2
		144	1.72±0.09	3.3±0.8	6.1±0.5	35.1±03	58.8±0.8	0.491±0.035	8.3±0.3	88.6±0.6	1.4±0.1	1.7±0.2
	2 mM 2-BrDA	72	1.62±0.11	7.1±1.1	5.5±0.3	34.0±0.7	60.5±0.4	0.265±0.081	9.6±0.3	87.6±0.6	1.3±0.1	1.5±0.2
		96	1.78±0.15	9.3±0.8	5.9±0.2	35.0±0.6	59.1±0.4	1.553±0.115	9.6±0.8	87.4±0.5	1.4±0.1	1.6±0.2
		120	1.82±0.15	9.5±0.9	6.3±0.2	35.2±0.5	58.5±0.7	1.801±0.079	9.3±0.3	87.7±0.6	1.4±0.1	1.6±0.2
		144	1.84±0.05	9.1±1.1	6.3±0.5	35.6±0.7	58.1±0.2	1.811±0.063	9.4±0.4	87.6±0.1	1.4±0.1	1.6±0.1

a Calculated from the GC data.

b 3HC, 3-hydroxyhexanoic acid; 3HO, 3-hydroxyoctanoic acid; 3HD, 3-hydroxydecanoic acid; 3HDDe, 3-hydroxy-*cis*-5-dodecenoic acid; 3HDD, 3-hydroxydodecanoic acid.

c Not detected by GC analysis.

RhlA and PhaG enzymes link *de novo* fatty acid synthesis to rhamnolipid and PHA synthesis, respectively [18], [21] (Figure 1). To investigate the independent role of 2-bromo-compounds on rhamnolipid and PHA production respectively, *P. aeruginosa* mutants for *phaG* and *rhlA* along with the corresponding wild-type bacteria were cultivated on 70 mM fructose in minimal medium. The transposon insertion mutant for *rhlA* from PA14 NR set (mutant ID 23291) [24] produced a significant amount of residual rhamnolipid, and was thus excluded from our experiments (data not shown). Instead we included the transposon insertion mutant of PAO1 for *rhlA* (PAO1-Δ*rhlA*) purchased for the parallel experiment in this study. The PAO1-Δ*rhlA* mutant grown with 70 mM fructose accumulated about 148% PHA compared to wild-type (Figure 3B). Here, the percent specific PHA content was defined by: 
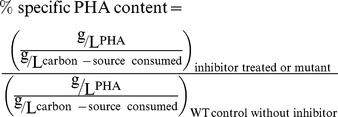
(1)


A possible explanation for this observation is that rhamnolipid synthesis enzymes may compete with the enzymes involved in PHA production for the consumption of the common fatty acid precursors [20]. Accordingly, PAO1-Δ*rhlA* displayed increased PHA accumulation, since fatty acid precursors were more available for PHA synthesis (Figure 1). However, upon addition of 2 mM 2-BrHA or 2-BrOA, PHA accumulation was dramatically decreased to 6.9 and 9.46% of that in wild-type, respectively (Figure 3B).

In the *phaG* mutant (PA14-Δ*phaG*) grown with 70 mM fructose, rhamnolipid synthesis was 76.1% of that of wild-type (Figure 3A). This result is in agreement with the previous report that the *phaG* mutant of *P. aeruginosa* strain ATCC 15692 showed a slight decrease in rhamnolipid production [40]. It is still unclear how and why the *phaG* mutant in *P. aeruginosa* strains shows a decrease in rhamnolipid production. As expected, addition of 2 mM 2-BrHA or 2-BrOA in 70 mM fructose significantly decreased rhamnolipid production to 3.1 and 7.5% that in wild-type, respectively (Figure 3A). However, 2-BrDA treatment led to only modest inhibition of rhamnolipid synthesis (69.6% of that in the wild-type). This specific inhibition of rhamnolipid and PHA synthesis strongly suggests that 2-BrHA and 2-BrOA may specifically and simultaneously target the two enzymes bridging the two pathways from fatty acid synthesis to rhamnolipid and PHA synthesis (Figure 1). The genome sequence of *P. aeruginosa* PA14 showed that RhlA and PhaG have about 57% sequence homology (41% identical) (data not shown). On the basis of the high sequence homology between RhlA and PhaG and the probable competition of both RhlA and PhaG against (R)-β-hydroxyalkanoyl-ACP, we considered the possibility that there may be dual targeting of 2-BrHA and 2-BrOA toward RhlA and PhaG. Similar to *P. fluorescens* BM07 [31], *P. aeruginosa* strains could not metabolize 2-BrOA (data not shown). A *Pseudomonas* sp. acyl-coenzyme A synthetase (E.C. 6.2.1.3) was not found to ligate CoA-SH to 2-BrOA whereas it synthesized (R)-β-hydroxydecanoyl-CoA from (R)-β-hydroxydecanoic acid and CoA-SH in the presence of ATP (unpublished work). Thus, free 2-bromo- compounds might be the inhibiting species. Further *in vitro* enzyme-level study of 2-bromo- compound-mediated inhibition of the PhaG enzyme was attempted for several years but was unsuccessful because of the failure (in our hands) of recombinant *Escherichia coli* derived protein to properly fold, in contrast to an earlier report [21].

### 2-BrHA and 2-BrOA inhibit the activity of PhaG and RhlA

**Figure 4:**
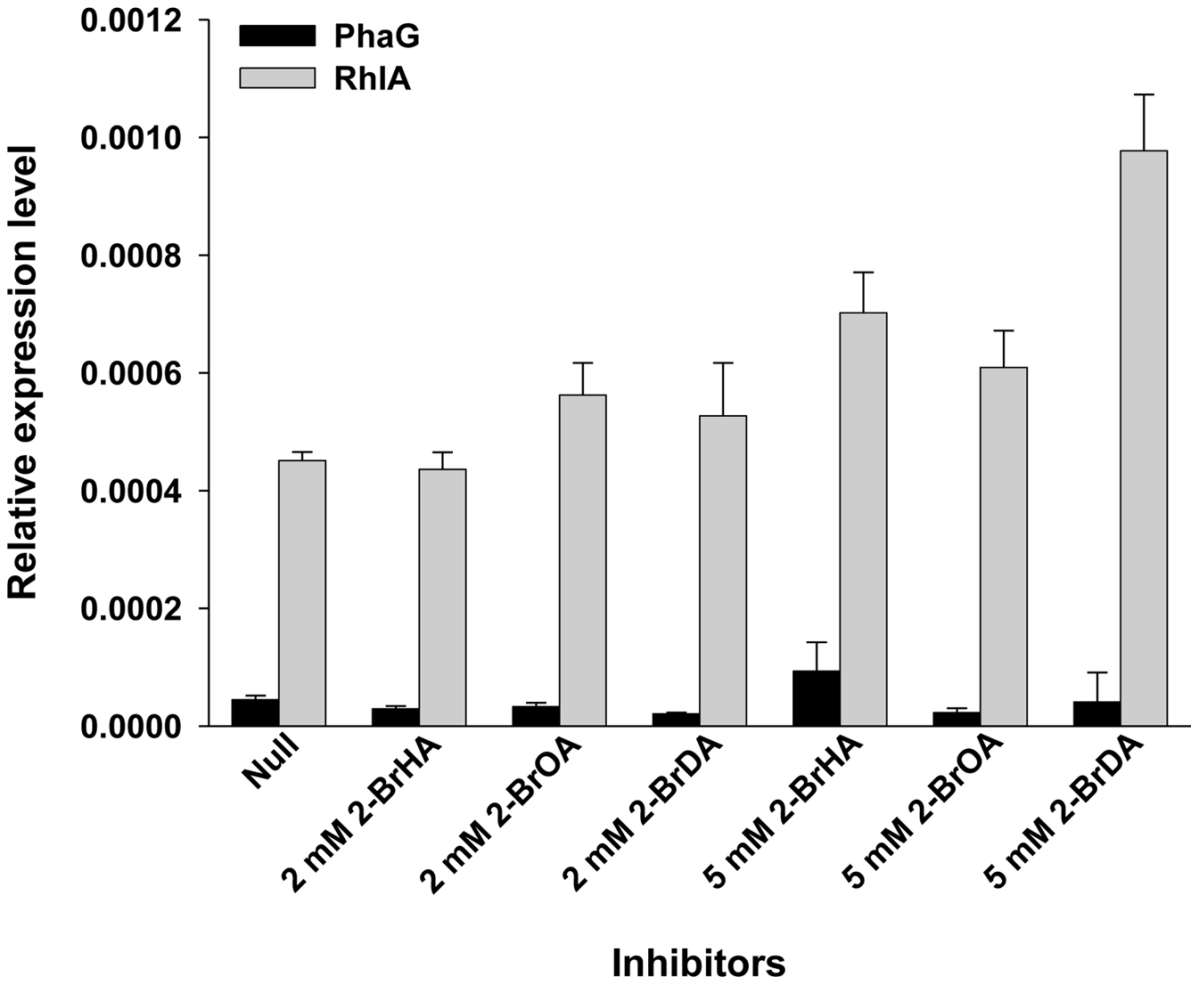
Effect of the 2-bromo-compounds 2-BrHA, 2-BrOA and 2-BrDA on the expression of the *phaG* and *rhlA* genes in the PAO1 strain. Cells were grown in M1 medium with 70 mM fructose in the presence or absence of 2 or 5 mM 2-bromo-compound at 30°C for 72 h.

To obtain any indirect evidence of the possibility of enzyme level inhibition, we investigated the effect of the 2-bromo- compounds on the expression of PhaG and RhlA in PAO1 wild-type (Figure 4). No suppression of the expression of the two genes was observed in the presence of 2∼5 mM 2-bromo- inhibitors. The 2-bromo- inhibitor independent expression of the genes *phaG* and *rhlA* may suggest a clue for the irrelevance of the inhibitors in transcriptional or/and translational activity. This supports the proposal that the bromo compounds inhibit the production of PHA and rhamnolipids by inhibiting the two enzymes PhaG and RhlA (Figure 1).

**Figure 5:**
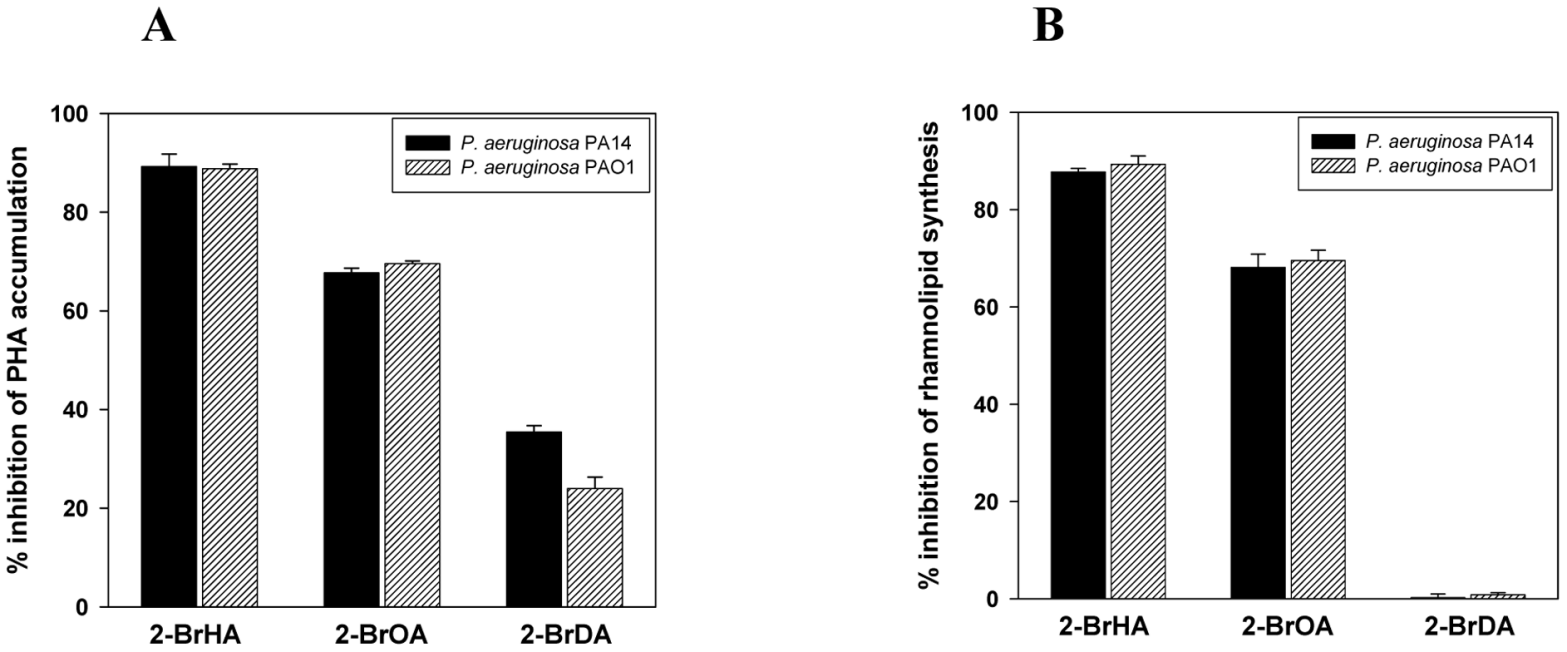
Inhibition strength of the three inhibitors 2-BrHA, 2-BrOA and 2-BrDA. Percent inhibition of PHA accumulation (A) and rhamnolipid synthesis (B) in *P. aeruginosa* PA14 and PAO1 grown in 70 mM fructose M1 medium.

For the probable dual targeting of the 2-bromo-compounds, additional evidence can be found in the comparative analysis of percent inhibition of PHA and rhamnolipid synthesis and biofilm formation depending on the alkyl chain length of the inhibitors (Figure 5). The two inhibitors 2-BrHA and 2-BrOA displayed similar levels of inhibition of PHA and rhamnolipid synthesis but 2-BrDA treatment resulted in a 25∼35% inhibition of PHA synthesis, but had little effect on rhamnolipid synthesis. The discrepancy observed for 2-BrDA treatment may imply that any gene upstream of *de novo* fatty acid synthesis is not the single target of the 2-bromo- inhibitors. Otherwise, 2-BrDA treatment should result in the same level of inhibition for the two products, just as occurred with 2-BrHA and 2-BrOA. The amino acid sequences of PAO1 RhlA and PA14 RhlA are 99% identical (they differ in that the Q in PA01 is replaced by P in PA14) and those of PAO1 PhaG and PA14 PhaG are 98% identical (PA14 PhaG differs from PAO1 PhaG in that residues A25, H74, E96, I154 and M215 of the latter are changed to S, R, D, V and V respectively). The amino acid sequences of PhaG and RhlA are 41% identical for both the PAO1 and PA14 strains. Thus, the high amino acid sequence homology of the two genes between the two strains may support the % inhibition data in Figure 5 as well as the view of dual targeting of the 2-bromo- compounds towards PhaG and RhlA.

### 2-Bromo- compounds inhibit swarming motility and biofilm formation

**Figure 6:**
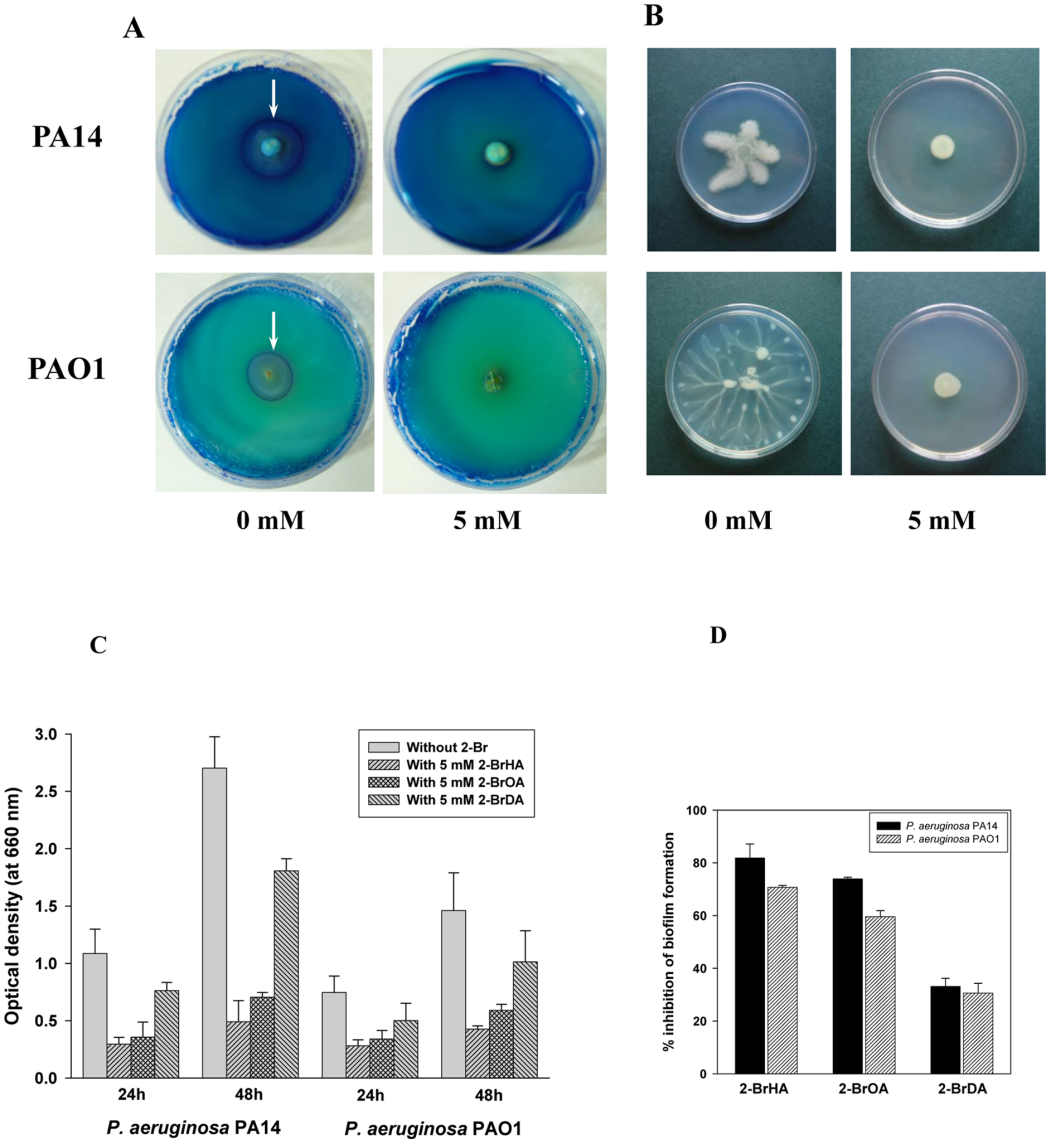
Effect of 2-BrHA, 2-BrOA and 2-BrDA on rhamnolipid production (blue halo test), swarming motility, and biofilm formation in *P. aeruginosa* strains PA14 and PAO1. (A) Rhamnolipid production (note the arrow) was estimated by a conventional agar plate assay (blue halo test, the result for 2-BrHA only is shown). (B) In the swarming motility assay, PA14 or PAO1 was spotted on plates with either none (0 mM) or 5 mM 2-BrHA and grown at 30°C for 48 h. (C) Biofilm formation was assayed using the crystal violet method. Each bar represents a standard deviation averaging three independent samples. (D) The % inhibition of biofilm formation in the PA14 and PAO1 strains, calculated from the data in (C).

*P. aeruginosa* is capable of performing a complex coordinated multicellular migration called ‘swarming’ [11]–[13], [41]. Swarming of *P. aeruginosa* is not only dependent on flagella but also on type IV pili [31]. Furthermore, rhamnolipids along with their precursor HAA (C_10_-C_10_), are known to be required for swarming motility based on the fact that *rhlA* mutants do not exhibit swarming motility, while *rhlB* and *rhlC* mutants are able to swarm [11]–[14]. The RhlA enzyme catalyzes synthesis of HAAs, which are converted to monorhamnolipids by the RhlB enzymes [12], [42]. Both the *rhlB* and *rhlC* genes encode rhamnosyltransferases that catalyze the formation of mono- and dirhamnolipids, respectively. Trembley et al. [14] reported that dirhamnolipids promote tendril formation and migration, thus acting as a self-produced chemotactic attractant, while HAAs play the opposite role, repelling swarming tendrils. Monorhamnolipids seem to act solely as a wetting agent that reduces the surface tension surrounding the colony. When grown on rhamnolipid plates, *P. aeruginosa* PA14 and PAO1 secreted detectable levels of rhamnolipids, as visualized by the blue halo in the plate assay (Figure 6A). In contrast, *P. aeruginosa* PA14 and PAO1 grown with 2-BrHA or 2-BrOA did not release detectable levels of rhamnolipid (only the data for 2-BrHA are shown). As expected, the cells grown with 2-BrHA or 2-BrOA displayed no swarming motility at all (Figure 6B) which is consistent with the inhibition of rhamnolipid production.

*P. aeruginosa* can also form diverse biofilms, which are cellular aggregates encased in an extracellular matrix consisting of protein, nucleic acids, exopolysaccharide and sometimes DNA, that holds the cells together in the community [43]. Surprisingly, treatment of the wild-type *P. aeruginosa* strains with the 2-bromo- compounds also inhibited biofilm formation prior to production of rhamnolipids (Figure 2 and Figure 6C and 6D). Differences in static biofilm formation were observed depending on the alkyl chain length of inhibitors when cells were grown on 70 mM fructose at 30°C. Growth of *P. aeruginosa* PA14 in the presence of 5 mM 2-BrHA, 2-BrOA and 2-BrDA for 48 h resulted in a decrease in biofilm formation to 18%, 26% and 67%, respectively, compared to the controls without 2-bromo- compounds (Figure 6C and 6D). For *P. aeruginosa* PAO1, addition of 5 mM 2-BrHA, 2-BrOA and 2-BrDA exhibited rather less significant suppression of biofilm formation, i.e. 29%, 40% and 69% at 48 h, respectively, compared to the controls. However, biofilm formation is known not to be related to rhamnolipid and PHA synthesis [44], [45]. Thus, further molecular level study is required to understand the role of the 2-bromo- compounds in inhibition.

### Metabolism of 2-bromo-fatty acids

**Figure 7:**
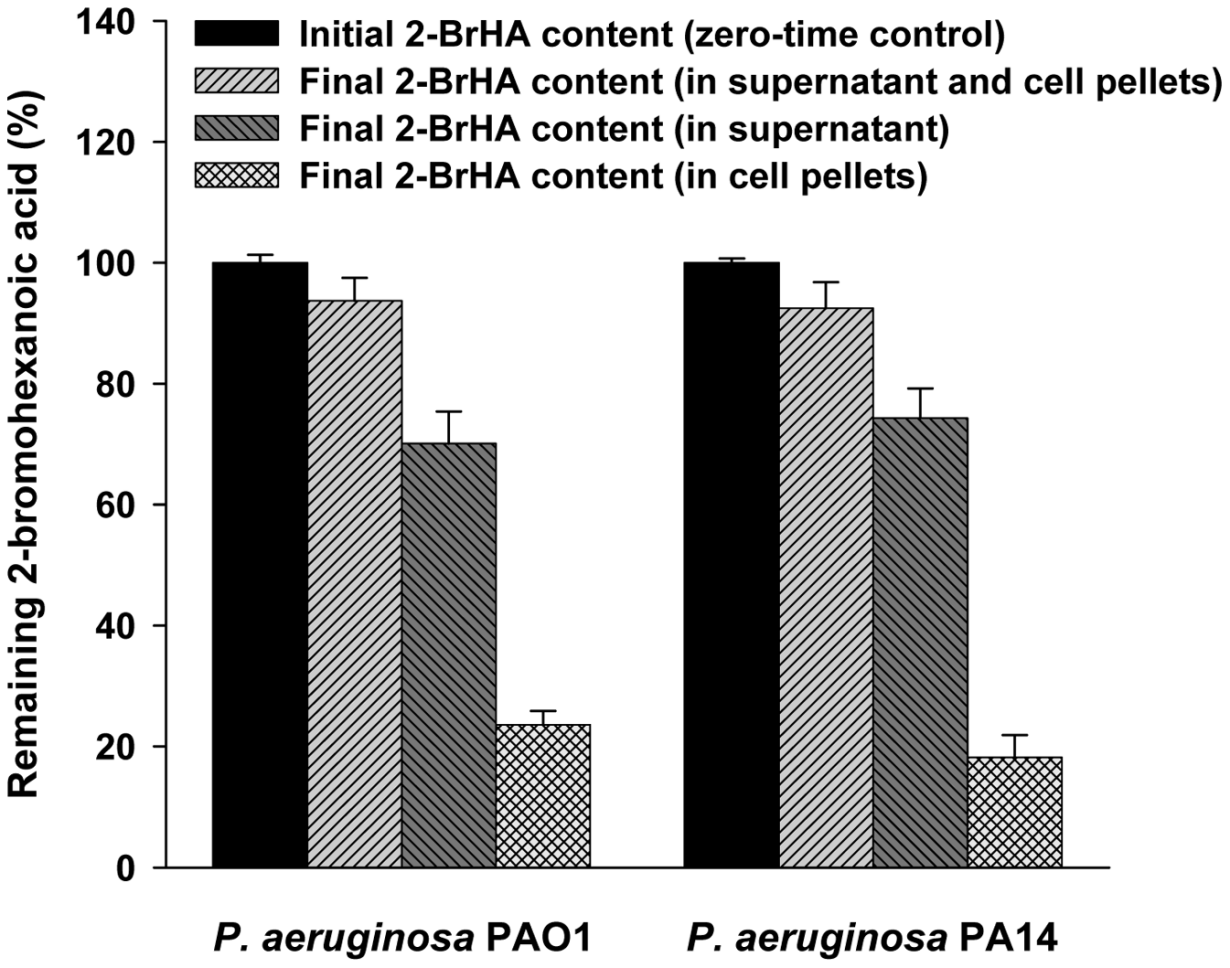
Amount of non-metabolized 2-BrHA remaining in the growth medium. The cells were grown in 70 mM fructose for 120 h. The initial concentration of 2-BrHA was 5 mM.

2-BrOA was reported not to be metabolized in *P. fluorescens* BM07 [33]. Similarly, all three 2-bromo-compounds when applied in combination with 70 mM fructose also were not metabolized in *P. aeruginosa*. Only the data for 2-BrHA are shown in Figure 7. Less than ∼7% of the inhibitor compounds in media seemed to be incorporated into the cells for both PAO1 and PA14 strains. Thus, millimolar (mM) level concentrations of the compounds were needed to obtain a substantial inhibitory effect. Actually, for 2-BrHA and 2-BrOA, concentrations of less than 300 µM induced little (less than 5%) inhibition on PHA and rhamnolipid production (data not shown). The *K_i_* for 50% inhibition was observed to be 60 µM for *P. fluorescens* BM07 [33]. It is unknown why such high concentrations of the inhibitors are required for *P. aeruginosa*.

In conclusion, our results indicate that the small molecule inhibitors 2-BrHA and 2-BrOA have the potential to control *P. aeruginosa* group behaviors as well as the syntheses of rhamnolipids and PHA. Thus, these 2-bromo- fatty acids which appear to inhibit multiple targets could be a valuable addition to *P. aeruginosa* group-behavior inhibitors for the development of anti-virulence compounds.
